# Effect of electroencephalography-based motor imagery neurofeedback on mu suppression during motor attempt in patients with stroke

**DOI:** 10.1186/s12984-025-01653-5

**Published:** 2025-05-28

**Authors:** Seungwoo Cha, Kyoung Tae Kim, Won Kee Chang, Nam-Jong Paik, Ji Soo Choi, Hyunmi Lim, Won-Seok Kim, Jeonghun Ku

**Affiliations:** 1 Department of Rehabilitation Medicine, University of Ulsan College of Medicine, Asan Medical Center, Seoul, Korea; 2 Department of Rehabilitation Medicine, Keimyung University School of Medicine, Keimyung University Dongsan Hospital, Daegu, Korea; 3 Department of Rehabilitation Medicine, Seoul National University College of Medicine, Seoul National University Bundang Hospital, Seongnam, Republic of Korea; 4 Department of Biomedical Engineering, School of Medicine, Keimyung University, Daegu, Korea

**Keywords:** Neurofeedback, Electroencephalography, Motor imagery, Mu suppression, Stroke, Upper extremity, Rehabilitation

## Abstract

**Objective:**

The primary aim of this study was to explore the neurophysiological effects of motor imagery neurofeedback using electroencephalography (EEG), specifically focusing on mu suppression during serial motor attempts, and to assess its potential benefits in patients with subacute stroke.

**Methods:**

A total of 15 patients with hemiplegia following subacute ischemic stroke were prospectively enrolled in this randomized cross-over study. This study comprised two experiments: neurofeedback and sham. Each experiment included four blocks: three blocks of resting, grasp, resting, and an interventional task, followed by one block of resting and grasp. During the resting sessions, participants fixated on a white cross on a black background for 2 min without moving their upper extremities. In the grasp sessions, participants were instructed to grasp and release their paretic hand at a frequency of about 1 Hz for 3 min while maintaining fixation on the white cross. During the interventional task, the neurofeedback presented a punching image using the affected upper limb, corresponding to the mu suppression induced by imagined movement for 3 min. In contrast, the sham presented an image based on mu suppression data from randomly selected participants. EEG data were recorded throughout the experiment, and data from electrodes C3/C4 and P3/P4 were analyzed to compare the degree of mu suppression between the neurofeedback and sham experiments.

**Results:**

Significant mu suppression was observed in the bilateral motor and parietal cortices during the neurofeedback experiment compared with the sham across serial sessions (*p* < 0.001). Following neurofeedback, real grasping sessions showed progressive strengthening of mu suppression in the ipsilesional motor cortex and bilateral parietal cortices compared to sessions following sham (*p* < 0.05). This effect was not observed in the contralesional motor cortex.

**Conclusions:**

Motor imagery neurofeedback significantly enhances mu suppression in the ipsilesional motor and bilateral parietal cortices during motor attempts in patients with subacute stroke. These findings suggest that motor imagery neurofeedback could serve as a promising adjunctive therapy to enhance motor-related cortical activity and support motor rehabilitation in patients with stroke.

**Supplementary Information:**

The online version contains supplementary material available at 10.1186/s12984-025-01653-5.

## Background

Stroke is a leading cause of death and disability. Although stroke mortality rates have decreased, the burden of stroke remains high, considering the growing and aging population [1]. Therefore, comprehensive and tailored rehabilitation strategies are gaining increasing attention. For stroke rehabilitation, organized stroke units, task-specific and repetitive training, goal setting and early supported discharge are recommended [2]. Recent technological advancements have spurred various efforts to enhance stroke rehabilitation.

Among these advances, neurofeedback using electroencephalography (EEG) or functional magnetic resonance imaging (MRI) has been explored in many studies. Neurofeedback is a biofeedback system in which neural activity is measured and presented in real-time to modulate the activity of targeted neural substrates [3]. A previous review demonstrated that neurofeedback can enhance motor recovery in patients with stroke when combined with conventional physical therapy, non-invasive brain stimulation, or robotic therapy [4]. As acquisition systems for neurofeedback, EEG with its high temporal resolution and functional MRI with its high spatial resolution are the two most representative methods. Notably, EEG has traditionally been used in many studies by leveraging the event-related desynchronization (ERD) of the mu rhythm or central beta rhythm [5].

Previous studies have reported somatotopic ERD patterns during motor imagination, which form the basis of EEG-based neurofeedback research [6]. Thereafter, paradigms for providing motor imagery neurofeedback through reliable ERD monitoring were proposed [7, 8]. Bartur et al. demonstrated that the magnitude of ERD in the motor cortex during motor attempt is directly proportional to the EMG activity of the paralyzed arm and inversely proportional to lesion size [9]. Additionally, a previous review highlighted that stroke recovery involves the reorganization of ERD during motor attempts, with changes observed in the ipsilesional and contralesional hemispheres [10]. Although previous research has explored the potential of motor imagery-based neurofeedback and established a link between ERD during motor attempts and stroke recovery, there is a paucity of studies examining how motor imagery-based neurofeedback specifically affects ERD during actual motor attempts. Moreover, considering that neurofeedback may serve as a potential adjunctive modality to enhance neuroplasticity during task-specific rehabilitation [11], further research is warranted to investigate how motor imagery-based neurofeedback influences ERD during real motion tasks. Understanding this relationship could provide valuable insights into the optimization of neurofeedback interventions to improve rehabilitation outcomes.

Therefore, our study aimed to investigate the impact of motor imagery-based neurofeedback on mu suppression in the sensorimotor cortex during the neurofeedback and subsequently during actual motor attempts in patients with unilateral upper limb weakness after subacute stroke. By assessing mu suppression across both phases, we sought to explore the potential effects of neurofeedback and its relevance to motor execution. We anticipate that the findings will provide mechanistic insights into how neurofeedback-induced changes during motor imagery may influence neuroplasticity during motor attempts and offer guidance for integrating neurofeedback into conventional task-based rehabilitation strategies.

## Methods

### Participants

Patients with hemiplegia after early subacute (from 1 week to 5 weeks) ischemic stroke were prospectively enrolled. Patients with cognitive impairment (Mini-Mental State Examination score < 16), delirium, depression, anxiety, or other uncontrolled medical conditions were excluded. Initially, 20 patients consented to participate and were enrolled in the study. However, three patients dropped out because of discomfort in maintaining posture and wearing the EEG; one patient was excluded as a result of an inability to follow instructions caused by decreased attention; and one patient was excluded as a result of poor EEG quality. Consequently, a total of 15 patients were included in the final analysis. This study was approved by the Institutional Review Board of Keimyung University Dongsan Hospital (IRB number 2021-04-112-003). All participants signed a written consent form.

### Study design

**Fig. 1:**
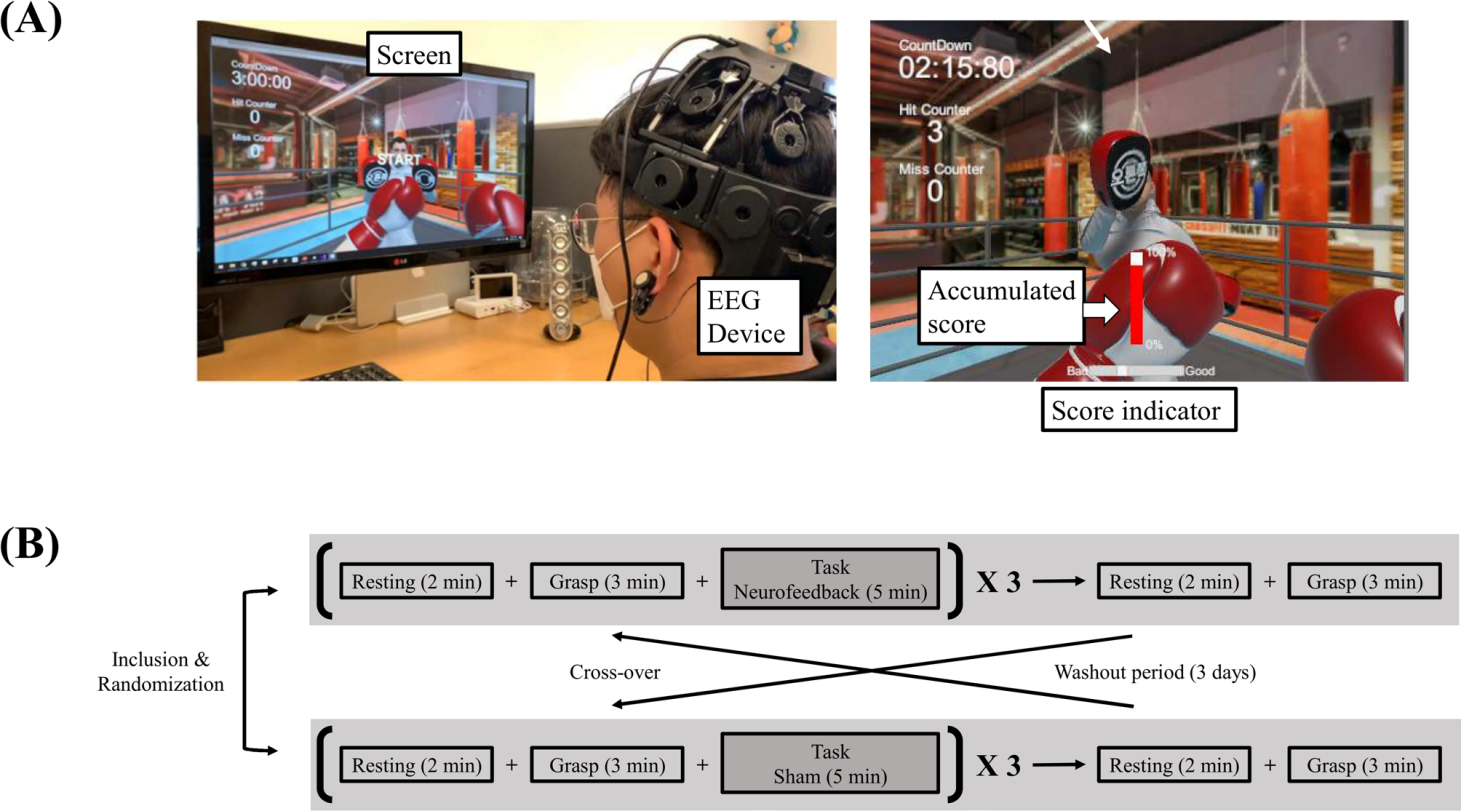
(**A**) A participant engaged in the neurofeedback intervention. (**B**) Study design

This randomized cross-over study consisted of two experiments: neurofeedback and sham (Fig. 1). Each experiment consisted of four blocks (three blocks of resting, grasp, resting, and an interventional task, followed by one block of resting and grasp). In the resting session, a white fixation cross on a black background appeared and remained fixed for 2 min, during which no upper extremity movement was allowed. In the grasp session, the same fixation cross appeared, and participants were instructed to grasp and release their weak hand at a frequency of about 1 Hz for 3 min. This was followed by another 2-min resting session. Then, participants spent 3 min imagining movements in the neurofeedback task session, during which a boxing image was generated based on mu suppression detected from the EEG. However, during the sham task session, EEG data recorded from another participant’s neurofeedback session were used to trigger the appearance of the boxing image on the screen instead of the participant’s real EEG. The first patient underwent neurofeedback first, with the sham condition using their own EEG. For subsequent patients, sham EEG data were randomly selected from the neurofeedback sessions of previous patients to generate a more realistic sham condition [12]. The two experiments were conducted in a randomized cross-over design with a washout period of at least 3 days─longer than the minute-level intervals used in previous studies [13, 14]─to reduce potential carry-over effects.

### Neurofeedback mechanism

The neurofeedback system is based on the asymmetry characteristics of mu suppression in the motor cortex. EEG data from the C3 and C4 channels were filtered using a fifth-order Butterworth bandpass filter allowing signals between 8 and 12 Hz. The EEG signal was then segmented into 1-s epochs, overlapping every 1/16 of a second, to calculate mu band power.

The mean and standard deviation of the mu power in C3 and C4 during a 1-min resting session were used to normalize the data. The normalized index was set such that a value of 0 corresponded to the mean mu power, + 1 corresponded to 2 standard deviations below the mean, and − 1 to 2 standard deviations above the mean. Thus, higher values reflected lower mu power, and lower values reflected higher mu power.

Normalized levels for the ipsilesional and contralesional hemispheres were obtained. The contrast score for each motor imagery training session was calculated by subtracting the contralesional index from the ipsilesional index. This score represented the relative strength of ipsilesional mu suppression. When the contrast score exceeded 0, it accumulated, and once it reached 2─an arbitrarily threshold─a punch to the target in the boxing game was triggered.

### EEG acquisition

EEG data were collected using 19 dry electrodes from the DSI-24 system (Wearable Sensing, San Diego, CA, USA). Electrodes were placed according to the International 10–20 system, and data were sampled at 300 Hz.

The EEG data were bandpass filtered between 4 and 30 Hz, and artifacts were rejected. Data were segmented into 2-s epochs. Epochs with an average amplitude exceeding 150 uV were excluded. Baseline correction and linear detrending were applied to each epoch. Mu band power was extracted using power spectral density analysis, and values were averaged. Mu band power during each grasp session was compared with the corresponding resting session. The degree of mu suppression was calculated using the log ratio. For EEG topography analysis, the lesion side was standardized to the left hemisphere: data from patients with left-sided lesions were used as-is, whereas data from patients with right-sided lesions were horizontally flipped.

### Statistical analysis

Mu suppression in the motor and parietal cortices was analyzed. Repeated-measures analysis of variance (ANOVA) for intervention (neurofeedback vs. sham) × time (from task 1 to task 3, or from grasp 1 to grasp 4), using a linear mixed-effects model, was performed to identify significant factors associated with mu suppression. Normality of model residuals was visually assessed using Q–Q plots for each condition. When statistical significance was detected for intervention or time, post-hoc comparisons between neurofeedback and sham, or between different time points, were conducted using Bonferroni-corrected *p*-values. Randomization order was included as a covariate in the model to control for potential order effects. Additional subgroup analyses were also performed. Statistical significance was set at *p* < 0.05. All analyses were conducted using R version 4.4.0 (R Foundation for Statistical Computing, Vienna, Austria).

## Results

A total of 15 patients with subacute cerebral infarctions were recruited for this study. The baseline characteristics of the study participants are presented in Table 1. The mean age of the participants was 66.5 years, and two were female. During the neurofeedback sessions, the average number of punches per 3-min session was 13.0 (95% confidence interval [CI]: 9.5–16.5) for task 1, 15.8 (95% CI: 12.3–19.3) for task 2, and 15.7 (95% CI: 13.2–18.1) for task 3. The average time required to generate a sufficient neurofeedback response per punch was 18.3 s (95% CI: 7.2–29.4) for task 1, 10.6 s (95% CI: 8.3–12.9) for task 2, and 10.0 s (95% CI: 8.3–11.8) for task 3. No statistically significant trends were observed in either the average number of punches or the average time required.

**Table 1:** Characteristics of patients with stroke included in the study

Number	Age (years)	Sex	Onset (days)	Laterality	Lesion location	mRS	FMA-UE	MMSE	MBI
1	80	Male	12	Right	Frontal	3	18	24	81
2	60	Male	15	Left	Corona radiata	3	66	29	80
3	56	Male	26	Right	Basal ganglia	4	7	25	15
4	82	Male	15	Right	Pons	4	61	17	59
5	79	Male	14	Left	Corona radiata	3	64	26	58
6	69	Male	13	Left	Corona radiata	4	46	28	38
7	49	Male	24	Right	Corona radiata	3	64	30	95
8	82	Female	9	Left	Corona radiata and Basal ganglia	4	51	19	37
9	53	Male	14	Right	Corona radiata	2	59	29	89
10	69	Male	12	Left	Thalamus	3	62	23	53
11	75	Male	19	Left	Medulla	3	64	23	44
12	40	Male	31	Left	Fronto-temporal and Temporo-occipital	2	65	25	97
13	83	Male	13	Right	Thalamus	4	60	22	38
14	50	Male	8	Right	Corona radiata and Basal ganglia	3	58	29	48
15	71	Female	24	Right	Pons	4	30	17	19

FMA-UE, Fugl-Meyer Assessment of the upper extremity; MBI, modified Barthel Index; MMSE, Mini-Mental State Examination; mRS, modified Rankin Scale

**Fig. 2:**
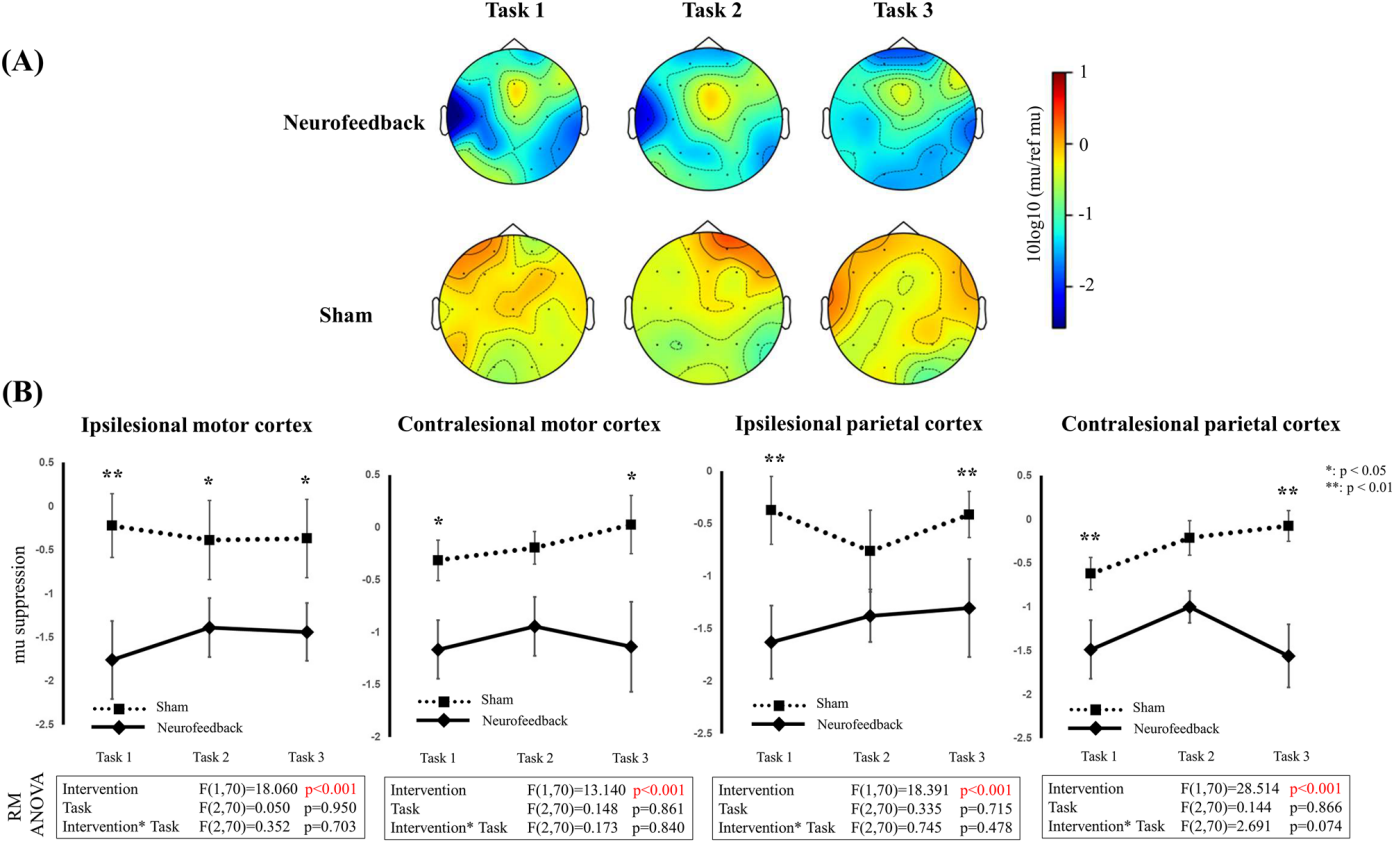
(**A**) Topography of mu suppression in electroencephalography during the intervention. The lesion side was standardized to the left hemisphere. (**B**) Line charts depicting mu suppression in the ipsilesional and contralesional motor cortices, as well as the ipsilesional and contralesional parietal cortices during the intervention. Results of repeated measures ANOVA are shown below the charts. Statistical significance from post-hoc analysis between neurofeedback and sham at each intervention is presented as *p < 0.05 or **p < 0.01

During the neurofeedback experiment, mu suppression was evident, whereas such suppression was not observed during the sham experiment (Fig. 2A). Figure 2B shows line charts of mu suppression in the ipsilesional and contralesional motor and parietal cortices. In the ipsilesional motor cortex, ANOVA indicated that the type of intervention was a significant factor influencing mu suppression (*p* < 0.001). Mu suppression was significantly greater during tasks 1, 2, and 3 in the neurofeedback (*p* = 0.002, 0.044, and 0.032, respectively) compared to the sham. In the contralesional motor cortex, ANOVA also indicated a significant effect of the intervention (*p* < 0.001), with greater mu suppression observed during tasks 1 and 3 in the neurofeedback (*p* = 0.039 and 0.014, respectively). In the ipsilesional parietal cortex, ANOVA showed a significant effect of intervention (*p* < 0.001), with significantly greater mu suppression in tasks 1 and 3 in the neurofeedback (*p* = 0.001 and 0.019, respectively). In the contralesional parietal cortex, ANOVA also showed a significant intervention effect (*p* < 0.001), with greater mu suppression in tasks 1 and 3 (*p* < 0.001 for both) in the neurofeedback.

**Fig. 3:**
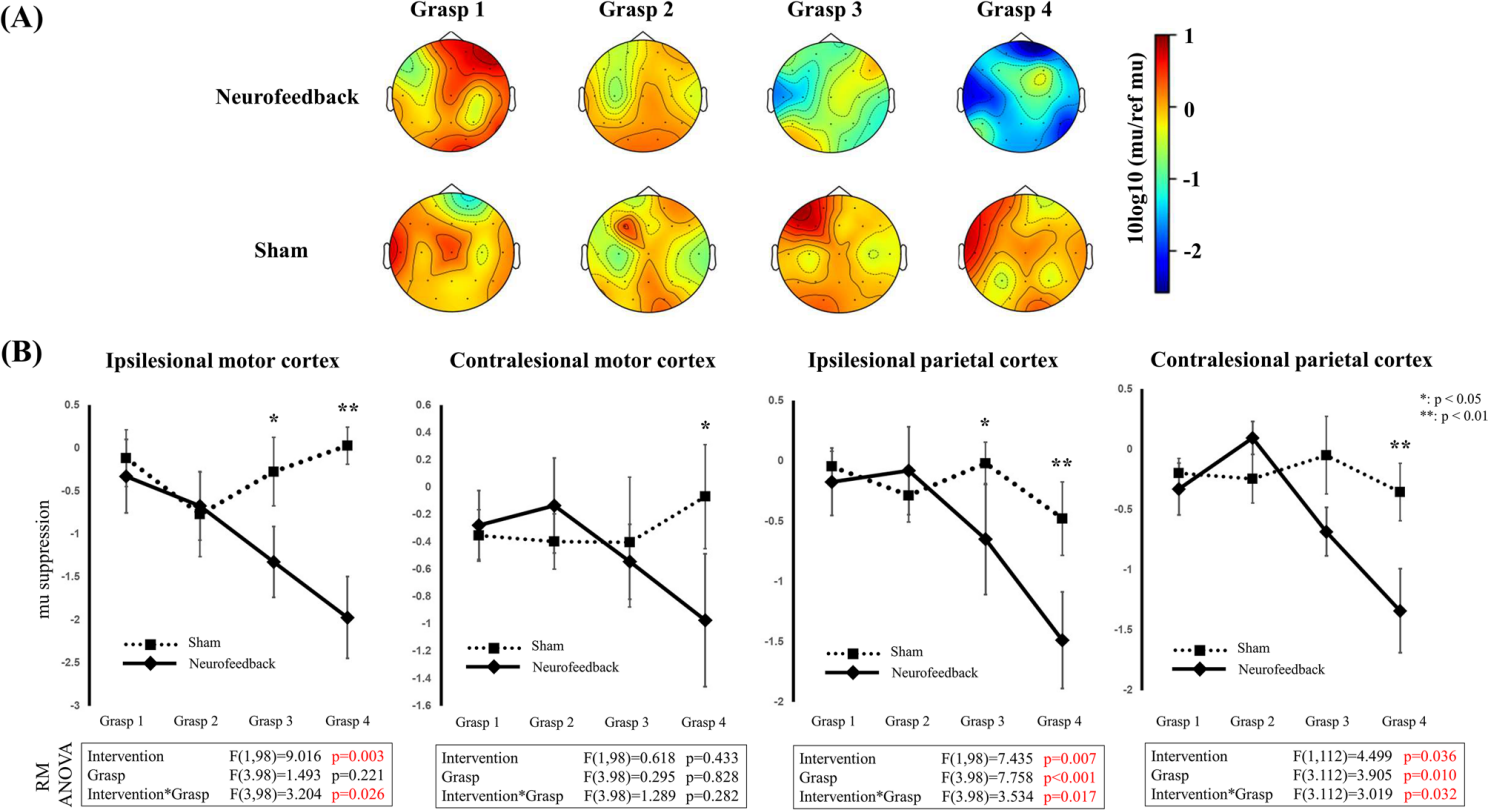
(**A**) Topography of mu suppression in electroencephalography during grasp. The lesion side was standardized to the left hemisphere. (**B**) Line charts depicting mu suppression in the ipsilesional and contralesional motor cortices, as well as the ipsilesional and contralesional parietal cortices during grasp. Results of repeated measures ANOVA are shown below the charts. Statistical significance from post-hoc analysis between neurofeedback and sham at each intervention is presented as *p < 0.05 or **p < 0.01

In the real grasp attempt session of the neurofeedback experiment, mu suppression increased gradually over time, whereas no significant change was observed in the sham grasp sessions. EEG topographies of each experiment are shown in Fig. 3A, and the corresponding line charts of mu suppression are shown in Fig. 3B. In the ipsilesional motor cortex, ANOVA showed that the type of intervention was a significant factor in mu suppression (*p* = 0.003), and a significant interaction between intervention and time was observed (*p* = 0.026). Post-hoc analysis showed that mu suppression in grasps 3 and 4 was significantly greater in the neurofeedback (*p* = 0.049 and *p* < 0.001, respectively) than in the sham. A significant difference was also found between grasps 1 and 4 in the neurofeedback (*p* = 0.014), whereas no such difference was observed in the sham. In the contralesional motor cortex, ANOVA did not show significant effects. In the ipsilesional parietal cortex, ANOVA showed significant main effects of intervention and time (*p* = 0.007 and 0.001, respectively), and a significant interaction (*p* = 0.017). Mu suppression in grasps 3 and 4 was significantly greater in the neurofeedback (*p* = 0.030 and *p* < 0.001, respectively). Significant differences in mu suppression were found between grasps 1 and 2, 1 and 3, and 1 and 4 (*p* < 0.001, < 0.001, and 0.024, respectively) in the neurofeedback, whereas no such differences were observed in the sham. In the contralesional parietal cortex, ANOVA showed significant effects of intervention, time, and their interaction (*p* = 0.036, 0.010, and 0.032, respectively). Mu suppression in grasp 4 was significantly greater in the neurofeedback (*p* = 0.003) than in the sham. Significant differences in mu suppression were found between grasps 1 and 4 and between grasps 2 and 4 (*p* = 0.018 and *p* < 0.001, respectively), only in the neurofeedback.

To evaluate the potential effect of randomization order (neurofeedback first or sham first), we re-analyzed the results using randomization order as a covariate and performed subgroup analysis. Although the effect of randomization order was not statistically significant in most conditions, it showed a marginally significant effect in the ipsilesional parietal cortex during the grasp (*p* = 0.047). In subgroup analysis, no substantial difference was observed in the overall trend between the two groups. Detailed statistical results are presented in Supplementary Tables 1 and 2.

**Fig. 4:**
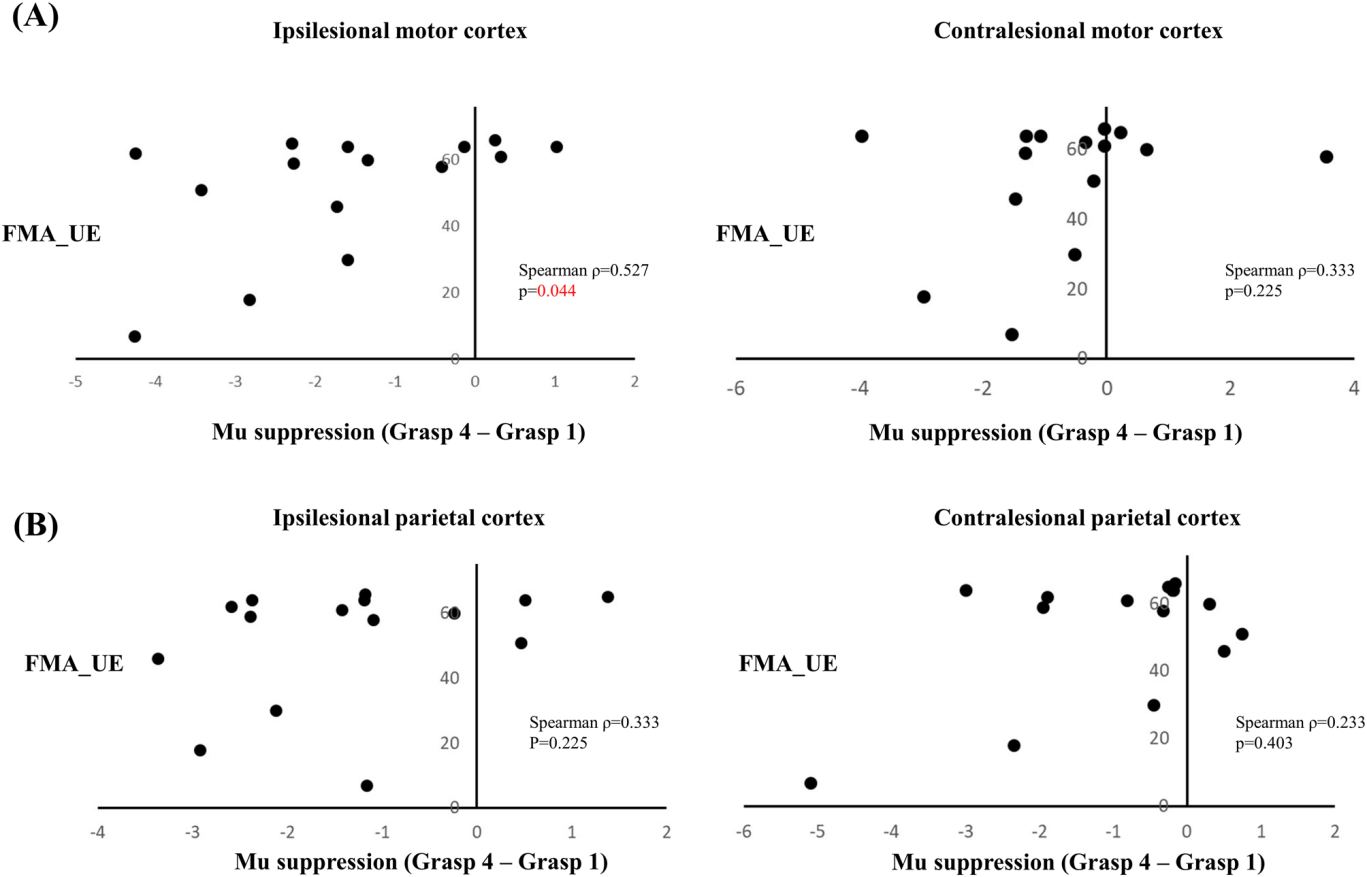
Scatter plots showing the relationship between the change in mu suppression (fourth grasp minus first grasp) and mu suppression in (**A**) ipsilesional and contralesional motor cortices and (**B**) ipsilesional and contralesional parietal cortices. Spearman correlation coefficients and *p*-values are presented

A moderate positive correlation was found between the Fugl–Meyer Assessment score of the upper extremity and the change in mu suppression between grasps 1 and 4 in the ipsilesional motor cortex (ρ = 0.527, *p* = 0.044) (Fig. 4A). No significant correlation was observed in the contralesional motor cortex or in either the ipsilesional or contralesional parietal cortices (Fig. 4A and B).

## Discussion

In this study, we investigated the effects of EEG-based motor imagery neurofeedback designed to enhance ipsilesional mu suppression in the sensorimotor cortex of patients with subacute stroke. Specifically, we observed that mu suppression in the motor and parietal cortices was significantly greater during the neurofeedback experiment. Furthermore, we found a progressive increase in mu suppression in the ipsilesional motor and bilateral parietal cortices during motor attempts following neurofeedback. Participants with lower Fugl-Meyer scores showed greater mu suppression in the ipsilesional motor cortex during the fourth grasp compared to the first. These findings provide important insights into the neurophysiology of neurofeedback training.

Similar to our study, a previous study using neurofeedback with mirror image revealed mu suppression in the ipsilesional motor cortex and parietal lobes of patients with stroke [13]. Another study using functional MRI showed that neurofeedback training increased the laterality of motor cortex activity, leading to improved hand motor performance as well as white matter tract changes [15]. Concordant with this, a meta-analysis found that neurofeedback intervention for post-stroke upper limb weakness was associated with the reorganization of cortical activity and clinical efficacy [16]. However, most previous studies focused on patients with chronic stroke, distinguishing our study by its focus on subacute stroke. Only a few studies, such as that by Hashimoto et al., have shown that neurofeedback with a brain-computer interface could be utilized successfully and safely in patients with acute stroke [17]. Considering that appropriate and tailored rehabilitation during the acute and subacute phases may affect long-term functional outcomes [18], more studies on the effects of EEG-based neurofeedback in patients with subacute stroke are warranted.

Previous studies have primarily monitored how mu suppression is observed on EEG during neurofeedback interventions. In contrast, our study is distinguished by examining mu suppression during grasp attempts following neurofeedback. This approach revealed that mu suppression during grasp in the ipsilesional primary motor cortex progressively increased with repeated motor imagery-based neurofeedback. In operant conditioning, learning happens when specific brain activity is matched with an external signal and reinforced by feedback when correct responses are made [19]. In this context, our findings suggest that motor imaginary neurofeedback may strengthen the ipsilesional sensorimotor loop, as in the study by Miller et al. [20], thereby facilitating motor recovery by re-establishing contingency.

Additionally, we observed enhanced mu suppression in the bilateral parietal cortices and the ipsilesional motor cortex during motor imagery-based neurofeedback, as well as during the subsequent execution of grasp activities. Previous studies have demonstrated that the bilateral parietal cortices play a critical role in motor imagination [21, 22] and are involved in motor intention or attempt [23, 24]. The involvement of the bilateral parietal cortices in both motor imagery and motor attempt underscores their importance in the neural processes underlying motor control. By leveraging their role in motor functions, neurofeedback interventions can be tailored to reinforce the neural pathways involved in motor recovery, potentially improving the efficacy of rehabilitation strategies for patients with stroke.

Interestingly, our study showed that a lower Fugl-Meyer score was associated with greater mu suppression during the fourth grasp than the first, suggesting that individuals with more moderate-to-severe weakness may benefit more from neurofeedback. Previous studies have shown that neurofeedback combined with brain-computer interfaces or robotic systems is feasible in patients with moderate-to-severe stroke, showing promising results [25–27]. Additionally, Kober et al. reported that patients with stroke exhibited changes in cortical activity on EEG following neurofeedback─changes that were not observed in a healthy control group [28]. These studies indicate that abnormal cortical activity patterns after stroke can be normalized through neurofeedback in patients with moderate-to-severe weakness. Conventional rehabilitation is typically based on repetitive training of volitional and task-specific movements. Therefore, patients with severe paralysis often experience challenges in eliciting responses or performing the required movements. Given these limitations, the use of neurofeedback in conjunction with conventional rehabilitation holds promise for providing more effective and personalized rehabilitation treatments.

This study has a few limitations. First, the neurofeedback intervention was brief and likely insufficient to elicit clinically meaningful effects. Furthermore, because videos of other participants undergoing neurofeedback were used as sham, it is possible that participants believed they were receiving neurofeedback, potentially making the sham act as an active control. No post-intervention interviews were conducted to determine whether participants believed they had received neurofeedback or sham. Although this sham approach may have reduced the observable differences between the groups, our study still found that mu suppression measured during grasping after neurofeedback was significantly different from sham in the ipsilesional motor cortex and bilateral parietal cortices. Finally, our study included a small number of participants with subacute stroke. Future research should provide neurofeedback to a larger cohort of patients over an extended period to evaluate its long-term effects.

## Conclusions

In our study, neurofeedback elicited significant mu suppression in the ipsilesional hemisphere of subacute stroke patients, which persisted during subsequent grasp tasks. This indicates that neurofeedback enhances motor-related cortical activity during real motor attempts, making it a promising addition to stroke rehabilitation. Our study also highlights the potential of neurofeedback to normalize abnormal brain activity and strengthen the ipsilesional sensorimotor loop. Future research involving a larger number of patients and long-term follow-up is needed to further elucidate its benefits and mechanisms.

## Electronic supplementary material

Below is the link to the electronic supplementary material.

Supplementary Material 1

## Data Availability

Pseudonymized data supporting the findings of this study are available from the corresponding author, Prof. Won-Seok Kim, upon reasonable request, subject to Institutional Review Board approval and completion of a legal data-sharing agreement.

## Abbreviations

ANOVA: Analysis of variance
CI: Confidence interval
ERD: Event-related desynchronization
MRI: Magnetic resonance imaging
EEG: Electroencephalography
