# Supplementary material for: Effect of electroencephalography-based motor imagery neurofeedback on mu suppression during motor attempt in patients with stroke
Source: J Neuroeng Rehabil. 2025 May 28;22:119. doi: 10.1186/s12984-025-01653-5 (PMC12117778; doi:10.1186/s12984-025-01653-5)
Supplement: Supplementary file 1 — Supplementary Material 1 [file 12984_2025_1653_MOESM1_ESM.docx]

**Supplementary Table 1.** Effect of randomization order on mu suppression.

(A) Randomization order as a covariable in the analysis of interventional task

|  | Ipsilesional motor cortex | | Contralesional motor cortex | | Ipsilesional parietal cortex | | Contralesional parietal cortex | |
| --- | --- | --- | --- | --- | --- | --- | --- | --- |
|  | F | p-value | F | p-value | F | p-value | F | p-value |
| Intervention | 18.06 | **< 0.001** | 13.14 | **< 0.001** | 18.39 | **< 0.001** | 28.51 | **< 0.001** |
| Task | 0.05 | 0.951 | 0.15 | 0.862 | 0.34 | 0.716 | 0.14 | 0.866 |
| Intervention*Task | 0.35 | 0.704 | 0.17 | 0.841 | 0.75 | 0.478 | 2.69 | 0.075 |
| Random | 0.65 | 0.436 | 1.65 | 0.221 | 0.05 | 0.828 | 0.001 | 0.971 |

(B) Randomization order as a covariable in the analysis of grasp

|  | Ipsilesional motor cortex | | Contralesional motor cortex | | Ipsilesional parietal cortex | | Contralesional parietal cortex | |
| --- | --- | --- | --- | --- | --- | --- | --- | --- |
|  | F | *p*-value | F | *p*-value | F | *p*-value | F | *p*-value |
| Intervention | 9.02 | **0.003** | 0.62 | 0.433 | 7.44 | **0.008** | 4.48 | **0.037** |
| Grasp | 1.49 | 0.221 | 0.3 | 0.829 | 7.76 | **< 0.001** | 3.89 | **0.011** |
| Intervention*Grasp | 3.2 | **0.027** | 1.29 | 0.282 | 3.53 | **0.018** | 3.01 | **0.034** |
| Random | 0.25 | 0.623 | 0.28 | 0.606 | 4.79 | **0.047** | 0.23 | 0.637 |

**Supplementary Table 2**. Subgroup analysis of mu suppression by randomization order.

(A) Mu suppression during interventional task in the neurofeedback-first group

|  | Ipsilesional motor cortex | | Contralesional motor cortex | | Ipsilesional parietal cortex | | Contralesional parietal cortex | |
| --- | --- | --- | --- | --- | --- | --- | --- | --- |
|  | F | *p*-value | F | *p*-value | F | *p*-value | F | *p*-value |
| Intervention | 12.61 | **0.001** | 7.58 | **0.009** | 7.86 | **0.009** | 14.8 | **0.001** |
| Task | 1.52 | 0.235 | 0.30 | 0.746 | 1.00 | 0.380 | 0.24 | 0.790 |
| Intervention*Task | 0.16 | 0.856 | 0.22 | 0.805 | 0.84 | 0.441 | 1.07 | 0.357 |

(B) Mu suppression during interventional task in the sham-first group

|  | Ipsilesional motor cortex | | Contralesional motor cortex | | Ipsilesional parietal cortex | | Contralesional parietal cortex | |
| --- | --- | --- | --- | --- | --- | --- | --- | --- |
|  | F | *p*-value | F | *p*-value | F | *p*-value | F | *p*-value |
| Intervention | 6.68 | **0.014** | 5.55 | **0.024** | 11.38 | **0.002** | 13.07 | **0.001** |
| Task | 1.22 | 0.301 | 0.01 | 0.989 | 0.31 | 0.732 | 0.42 | 0.663 |
| Intervention*Task | 0.55 | 0.582 | 0.77 | 0.471 | 0.15 | 0.862 | 1.50 | 0.237 |

(C) Mu suppression during grasp in the neurofeedback-first group

|  | Ipsilesional motor cortex | | Contralesional motor cortex | | Ipsilesional parietal cortex | | Contralesional parietal cortex | |
| --- | --- | --- | --- | --- | --- | --- | --- | --- |
|  | F | *p*-value | F | *p*-value | F | *p*-value | F | *p*-value |
| Intervention | 5.59 | **0.023** | 0.66 | 0.423 | 2.89 | 0.096 | 1.23 | 0.274 |
| Grasp | 1.40 | 0.255 | 0.32 | 0.809 | 8.02 | **< 0.001** | 0.94 | 0.428 |
| Intervention*Grasp | 2.33 | 0.088 | 0.78 | 0.510 | 2.37 | 0.084 | 0.27 | 0.844 |

(D) Mu suppression during grasp in the sham-first group

|  | Ipsilesional motor cortex | | Contralesional motor cortex | | Ipsilesional parietal cortex | | Contralesional parietal cortex | |
| --- | --- | --- | --- | --- | --- | --- | --- | --- |
|  | F | *p*-value | F | *p*-value | F | *p*-value | F | *p*-value |
| Intervention | 3.44 | 0.070 | 0.07 | 0.794 | 4.47 | **0.040** | 3.37 | 0.072 |
| Grasp | 0.55 | 0.652 | 1.14 | 0.343 | 1.72 | 0.176 | 3.57 | **0.020** |
| Intervention*Grasp | 1.23 | 0.309 | 1.10 | 0.357 | 1.31 | 0.282 | 3.62 | **0.018** |
